# Supplementary material for: Serological Evidence for Circulation of Influenza D Virus in the Ovine Population in Italy
Source: Pathogens. 2024 Feb 11;13(2):162. doi: 10.3390/pathogens13020162 (PMC10892703; doi:10.3390/pathogens13020162)
Supplement: Supplementary file 1 [file pathogens-13-00162-s001.zip › pathogens-2846475-supplementary.pdf]

Table S1: Average viral titers obtained using influenza D/bovine/Oklahoma/660/2013 (D/660) and D/swine/Italy/199724/2015 (D/OK) strains by haemagglutination inhibition (HI) and/or virus neutralization (VN) assays

Sample	Sheep Herd	Prefecture	HI		VN	
			D/660	D/OK	D/660	D/OK
1	A	RG	5	5	20	5
2	A	RG	5	5	20	5
3	A	RG	5	5	20	5
4	A	RG	5	5	20	5
5	A	RG	5	5	20	5
6	A	RG	5	5	28	5
7	A	RG	5	5	5	5
8	A	RG	5	5	20	5
9	A	RG	5	5	40	5
10	A	RG	5	5	20	20
11	A	RG	5	5	5	5
12	A	RG	5	5	14	5
13	A	RG	5	5	5	5
14	A	RG	5	5	20	5
15	A	RG	5	5	20	28
16	A	RG	5	5	20	5
17	A	RG	5	5	5	5
18	A	RG	5	5	5	5
19	A	RG	5	5	10	5
20	A	RG	5	5	20	5
21	B	SR	5	5	14	5
22	B	SR	5	5	5	5
23	B	SR	5	5	10	5
24	B	SR	5	5	40	20
25	B	SR	5	5	10	10
26	B	SR	5	5	14	20
27	B	SR	5	5	20	5
28	B	SR	5	5	40	5
29	B	SR	5	5	5	5
30	B	SR	5	10	10	28
31	B	SR	5	5	20	5
32	B	SR	10	5	40	5
33	B	SR	5	5	14	5
34	B	SR	5	5	20	5
35	B	SR	5	5	5	5
36	B	SR	5	5	20	40
37	B	SR	10	5	40	14
38	B	SR	5	5	5	5
39	B	SR	5	5	5	5
40	B	SR	28	5	80	5
41	B	SR	5	5	20	5
42	B	SR	5	5	40	28
43	B	SR	5	5	5	5

44	B	SR	5	5	20	5
45	B	SR	5	5	20	5
46	B	SR	5	5	5	5
47	B	SR	5	5	28	5
48	B	SR	5	5	57	5
49	B	SR	5	5	14	5
50	B	SR	5	5	14	5
51	B	SR	5	5	10	5
52	B	SR	5	5	14	5
53	B	SR	5	5	28	5
54	B	SR	5	5	5	5
55	B	SR	5	5	5	5
56	B	SR	5	5	40	5
57	B	SR	5	5	20	5
58	B	SR	5	5	5	5
59	B	SR	5	5	20	5
60	B	SR	5	10	10	57
61	B	SR	5	5	20	5
62	B	SR	5	5	28	5
63	B	SR	5	5	5	5
64	B	SR	5	5	14	20
65	B	SR	5	5	5	5
66	B	SR	5	5	20	5
67	B	SR	5	5	10	5
68	B	SR	5	5	20	5
69	B	SR	5	5	14	5
70	B	SR	5	5	5	5
71	B	SR	5	5	14	5
72	B	SR	5	5	80	5
73	B	SR	5	5	160	5
74	B	SR	5	5	20	5
75	B	SR	5	5	40	5
76	B	SR	5	5	5	5
77	B	SR	5	5	28	5
78	C	SR	5	5	5	5
79	C	SR	5	5	40	5
80	C	SR	5	5	20	5
81	C	SR	5	5	28	5
82	C	SR	5	5	28	5
83	C	SR	5	5	80	5
84	C	SR	5	5	57	5
85	A	RG	5	5	20	5
86	A	RG	5	5	5	5
87	A	RG	20	5	80	20
88	A	RG	10	10	5	5
89	A	RG	20	5	40	5
90	A	RG	10	5	28	5
91	A	RG	10	5	5	5
92	A	RG	5	5	40	5

93	A	RG	28	5	40	5
94	A	RG	40	20	57	226
95	A	RG	5	5	14	5
96	A	RG	5	5	5	5
97	A	RG	14	5	40	5
98	A	RG	10	10	28	40
99	A	RG	10	5	28	5
100	A	RG	5	5	5	5
101	A	RG	5	5	5	5
102	A	RG	10	40	5	113
103	A	RG	5	5	20	28
104	A	RG	5	5	57	5
105	A	RG	20	5	57	5
106	A	RG	5	5	28	5
107	A	RG	5	5	5	5
108	A	RG	5	5	5	5
109	A	RG	5	5	40	5
110	A	RG	5	5	5	5
111	A	RG	5	5	5	5
112	A	RG	5	5	5	5
113	A	RG	5	5	160	5
114	A	RG	5	5	5	5
115	D	RG	5	10	40	28
116	D	RG	5	5	80	5
117	D	RG	5	5	40	5
118	D	RG	5	5	5	5
119	D	RG	5	5	20	5
120	D	RG	5	5	5	5
121	D	RG	5	5	40	5
122	D	RG	5	5	40	5
123	D	RG	5	5	57	5
124	D	RG	5	5	5	5
125	D	RG	5	5	5	5
126	D	RG	5	5	5	5
127	D	RG	5	5	5	5
128	D	RG	5	5	5	5
129	D	RG	5	5	80	5
130	D	RG	5	5	5	5
131	D	RG	28	5	57	5
132	D	RG	5	5	5	5
133	D	RG	5	5	5	5
134	D	RG	5	5	5	5
135	D	RG	10	5	57	40
136	D	RG	10	5	57	14
137	D	RG	5	5	28	5
138	D	RG	5	5	5	5
139	D	RG	5	5	5	5
140	D	RG	14	5	40	5
141	D	RG	5	5	5	5

142	D	RG	5	5	57	5
143	D	RG	5	5	20	5
144	D	RG	5	5	14	5
145	D	RG	5	5	5	5
146	D	RG	5	5	5	5
147	D	RG	5	5	20	5
148	D	RG	5	5	5	5
149	D	RG	5	5	20	5
150	D	RG	5	5	80	5
151	D	RG	5	5	28	5
152	D	RG	5	5	5	5
153	D	RG	5	5	5	5
154	D	RG	5	5	5	5
155	D	RG	10	5	160	5
156	D	RG	5	5	40	5
157	D	RG	10	5	40	5
158	D	RG	5	5	5	5
159	D	RG	10	5	160	5
160	D	RG	5	5	5	5
161	D	RG	5	5	5	5
162	D	RG	5	5	5	5
163	D	RG	5	5	5	5
164	D	RG	5	5	28	5
165	D	RG	5	5	5	5
166	D	RG	5	5	5	5
167	D	RG	5	5	10	5
168	D	RG	5	5	28	5
169	D	RG	5	5	40	5
170	D	RG	5	5	160	5
171	D	RG	5	5	28	10
172	D	RG	5	5	40	5
173	D	RG	5	5	5	5
174	D	RG	5	5	28	5
175	E	RG	5	5	5	5
176	E	RG	5	5	20	5
177	E	RG	5	5	40	5
178	E	RG	5	5	5	5
179	E	RG	40	5	113	5
180	E	RG	20	5	113	5
181	E	RG	5	5	5	5
182	E	RG	5	5	5	5
183	E	RG	5	5	5	5
184	E	RG	28	5	226	5
185	E	RG	5	5	28	5
186	E	RG	14	5	28	40
187	E	RG	5	5	40	5
188	E	RG	5	5	20	5
189	E	RG	20	5	20	5
190	E	RG	20	5	160	5

191	E	RG	10	5	57	5
192	E	RG	5	5	40	5
193	E	RG	5	5	5	5
194	E	RG	10	5	57	5
195	E	RG	14	10	5	28
196	E	RG	20	5	57	5
197	E	RG	5	5	5	5
198	E	RG	5	5	5	5
199	E	RG	5	5	5	5
200	E	RG	5	5	5	5
201	E	RG	5	5	5	5
202	E	RG	10	5	80	5
203	E	RG	5	5	5	5
204	E	RG	20	10	160	28
205	E	RG	5	5	80	5
206	E	RG	5	5	5	5
207	E	RG	10	5	20	10
208	E	RG	14	5	20	5
209	E	RG	14	5	160	5
210	E	RG	5	5	5	5
211	E	RG	5	5	5	5
212	E	RG	5	10	28	40
213	E	RG	5	5	5	5
214	E	RG	5	5	5	5
215	F	RG	5	5	20	5
216	F	RG	10	5	20	5
217	F	RG	5	5	28	5
218	F	RG	5	5	5	5
219	F	RG	5	5	5	5
220	F	RG	5	5	5	5
221	F	RG	14	5	80	5
222	F	RG	10	5	80	5
223	F	RG	10	10	40	28
224	F	RG	20	10	40	40
225	F	RG	5	5	40	5
226	F	RG	10	5	40	5
227	F	RG	20	5	28	5
228	F	RG	5	5	5	5
229	F	RG	5	5	5	5
230	F	RG	5	5	5	5
231	F	RG	5	5	5	5
232	F	RG	5	5	5	5
233	F	RG	5	5	57	5
234	F	RG	5	5	5	5
235	F	RG	5	5	5	5
236	F	RG	5	5	5	5
237	F	RG	5	5	5	5
238	F	RG	5	5	5	5
239	F	RG	5	5	5	5

240	F	RG	5	5	5	5
241	F	RG	5	5	14	14
242	F	RG	5	5	5	5
243	F	RG	5	5	5	5
244	F	RG	10	5	40	14
245	F	RG	5	5	5	5
246	F	RG	5	5	5	5
247	F	RG	5	5	5	5
248	F	RG	40	5	80	5
249	F	RG	10	5	80	5
250	F	RG	5	5	5	5
251	F	RG	5	5	5	5
252	F	RG	20	5	40	5
253	F	RG	5	5	5	5
254	F	RG	5	5	5	5
255	F	RG	5	5	5	5
256	F	RG	5	5	5	5
257	F	RG	5	5	5	5
258	D	RG	10	5	40	5
259	D	RG	5	5	5	5
260	D	RG	5	5	5	5
261	D	RG	5	5	5	5
262	D	RG	5	5	20	5
263	G	SR	5	5	28	5
264	G	SR	5	5	40	5
265	G	SR	5	5	5	5
266	G	SR	5	10	10	40
267	G	SR	5	5	5	5
268	G	SR	5	5	5	5
269	G	SR	5	5	40	5
270	G	SR	5	5	40	10
271	G	SR	5	5	5	5
272	G	SR	5	5	5	5
273	G	SR	5	5	5	5
274	G	SR	20	5	40	5
275	G	SR	5	5	5	5
276	G	SR	5	5	5	5
277	G	SR	5	5	14	5
278	G	SR	10	20	40	14
279	G	SR	5	5	40	20
280	G	SR	5	5	5	5
281	G	SR	5	5	5	5
282	G	SR	5	5	20	5
283	G	SR	5	5	80	5
284	G	SR	40	10	57	10
285	G	SR	5	10	20	20
286	G	SR	5	5	20	5
287	G	SR	5	5	20	5
288	G	SR	5	5	5	5

289	G	SR	5	5	5	5
290	G	SR	20	40	160	160
291	G	SR	5	5	5	5
292	G	SR	5	5	5	5
293	G	SR	5	5	5	5
294	G	SR	5	5	40	5
295	G	SR	5	5	5	5
296	G	SR	5	5	5	5
297	G	SR	5	5	5	5
298	G	SR	10	5	40	5
299	G	SR	5	5	5	5
300	G	SR	5	5	5	5
301	G	SR	14	5	57	10
302	G	SR	5	5	20	5
303	G	SR	5	5	80	5
304	G	SR	10	5	40	5
305	G	SR	5	5	5	5
306	G	SR	5	5	5	5
307	G	SR	14	5	40	5
308	G	SR	5	5	5	5
309	G	SR	5	5	40	5
310	G	SR	14	5	80	5
311	G	SR	5	5	20	5
312	G	SR	5	5	14	5
313	H	SR	5	10	20	28
314	H	SR	5	5	28	5
315	H	SR	5	5	5	5
316	H	SR	5	5	5	5
317	H	SR	5	5	40	5
318	H	SR	5	5	14	5
319	H	SR	5	5	28	5
320	H	SR	5	5	5	5
321	H	SR	5	5	57	5
322	H	SR	5	14	28	28
323	H	SR	5	5	5	10
324	H	SR	5	5	40	20
325	E	RG	5	5	5	5
326	E	RG	5	5	5	5
327	E	RG	5	5	28	5
328	E	RG	5	5	20	5
329	E	RG	5	5	5	5
330	E	RG	5	5	20	5
331	E	RG	20	5	20	5
332	E	RG	5	5	40	14
333	E	RG	14	10	40	28
334	E	RG	5	5	5	5
335	E	RG	5	5	5	5
336	E	RG	14	10	80	28
337	E	RG	10	5	40	5

338	E	RG	10	5	28	5
339	E	RG	5	5	5	5
340	E	RG	5	5	5	5
341	E	RG	20	5	57	5
342	E	RG	20	5	80	28
343	E	RG	5	5	160	5
344	E	RG	5	5	28	5
345	E	RG	5	5	14	5
346	E	RG	5	5	5	5
347	E	RG	5	5	20	5
348	E	RG	5	5	5	5
349	I	RG	5	5	40	5
350	I	RG	5	5	20	5
351	I	RG	10	5	5	5
352	I	RG	5	5	5	5
353	I	RG	5	5	28	10
354	I	RG	5	5	5	5
355	I	RG	10	5	28	5
356	I	RG	10	5	40	5
357	I	RG	5	10	5	113
358	I	RG	5	5	5	5
359	I	RG	5	5	40	5
360	I	RG	5	5	5	5
361	I	RG	20	5	40	5
362	I	RG	5	5	5	5
363	I	RG	5	5	5	5
364	I	RG	14	5	20	28
365	I	RG	20	5	40	28
366	I	RG	20	5	80	5
367	I	RG	5	5	5	5
368	I	RG	5	5	5	5
369	I	RG	80	5	226	5
370	I	RG	5	5	5	5
371	I	RG	5	5	20	5
372	I	RG	5	5	5	5
373	I	RG	5	5	5	5
374	I	RG	20	5	28	5
375	I	RG	5	5	5	5
376	I	RG	5	5	5	5
377	I	RG	20	5	40	5
378	I	RG	5	5	5	5
379	I	RG	5	5	5	5
380	I	RG	5	5	5	5
381	I	RG	5	5	20	5
382	I	RG	5	5	5	5
383	I	RG	5	5	5	5
384	I	RG	5	5	40	5
385	I	RG	5	5	20	5
386	I	RG	5	5	113	5

387	I	RG	5	5	5	5
388	I	RG	5	5	5	5
389	I	RG	20	5	40	28
390	I	RG	14	5	57	5
391	I	RG	10	5	40	5
392	I	RG	40	5	160	5
393	I	RG	5	5	5	5
394	I	RG	5	5	5	5
395	I	RG	5	5	5	5
396	I	RG	5	5	5	5
397	I	RG	5	5	20	5
398	I	RG	10	5	40	5
399	I	RG	5	5	40	5
400	I	RG	10	5	28	14
401	I	RG	5	5	5	5
402	I	RG	5	5	5	5
403	I	RG	40	5	160	20
404	I	RG	20	5	40	57
405	I	RG	20	5	40	5
406	I	RG	10	5	20	5
407	I	RG	5	5	5	5
408	I	RG	10	5	20	5
409	I	RG	28	5	40	5
410	I	RG	20	5	5	40
411	I	RG	20	5	40	40
412	I	RG	5	5	20	10
413	I	RG	5	5	5	5
414	I	RG	14	5	57	5
415	I	RG	10	5	20	14
416	I	RG	10	5	20	28
417	I	RG	40	5	80	28
418	I	RG	80	5	160	57
419	G	SR	5	5	5	5
420	G	SR	5	5	5	40
421	G	SR	5	5	28	5
422	G	SR	5	5	40	5
423	G	SR	5	5	5	5
424	G	SR	5	5	5	20
425	G	SR	10	5	80	20
426	J	SR	5	5	5	5
427	J	SR	5	5	5	5
428	J	SR	5	5	5	20
429	J	SR	5	5	5	20
430	J	SR	5	5	5	5
431	J	SR	20	40	453	453
432	J	SR	5	5	5	5
433	J	SR	20	5	14	28
434	J	SR	5	5	20	20
435	J	SR	160	5	320	40

436	J	SR	40	10	160	80
437	J	SR	5	5	5	5
438	J	SR	5	5	5	5
439	J	SR	5	5	40	5
440	J	SR	5	5	5	5
441	J	SR	5	5	5	20
442	J	SR	5	5	20	5
443	J	SR	10	5	40	57
444	J	SR	28	5	160	40
445	J	SR	5	5	5	5
446	J	SR	5	5	5	5
447	J	SR	160	20	1280	320
448	J	SR	5	5	10	5
449	J	SR	5	5	5	5
450	J	SR	5	5	14	20
451	J	SR	20	5	113	113
452	J	SR	5	5	80	5
453	J	SR	14	5	5	5
454	J	SR	20	5	40	20
455	J	SR	5	5	5	5
456	J	SR	5	5	5	5
457	J	SR	10	5	20	5
458	J	SR	20	5	57	40
459	J	SR	5	5	5	5
460	J	SR	5	5	5	5
461	J	SR	5	5	40	20
462	J	SR	28	5	80	57
463	J	SR	20	10	113	113
464	J	SR	40	5	640	57
465	J	SR	57	5	160	57
466	J	SR	5	5	40	57
467	J	SR	14	5	80	57
468	J	SR	10	5	40	5
469	J	SR	14	5	80	20
470	J	SR	10	5	5	5
471	K	RG	5	5	5	5
472	K	RG	5	5	40	20
473	K	RG	5	5	5	5
474	K	RG	5	5	80	5
475	K	RG	5	5	20	5
476	K	RG	5	5	5	5
477	K	RG	5	5	20	40
478	K	RG	5	5	14	5
479	K	RG	10	5	40	40
480	K	RG	5	5	40	5
481	K	RG	20	5	57	20
482	K	RG	40	10	113	57
483	K	RG	10	5	57	5
484	K	RG	10	5	57	28

485	K	RG	10	5	5	5
486	K	RG	10	5	57	14
487	K	RG	20	5	40	40
488	K	RG	5	5	5	5
489	K	RG	10	5	40	5
490	K	RG	5	5	28	40
491	K	RG	5	5	10	10
492	K	RG	5	5	10	5
493	K	RG	20	5	57	80
494	K	RG	5	5	20	5
495	K	RG	5	5	5	5
496	K	RG	20	5	5	5
497	K	RG	10	5	40	5
498	K	RG	5	5	20	5
499	K	RG	5	5	10	5
500	K	RG	5	5	10	5
501	K	RG	10	5	14	5
502	K	RG	20	5	80	80
503	K	RG	20	5	80	5
504	K	RG	5	5	14	5
505	K	RG	5	5	10	5
506	K	RG	20	5	57	5
507	K	RG	5	5	20	5
508	K	RG	5	5	5	5
509	K	RG	5	5	5	5
510	K	RG	5	5	20	5
511	K	RG	20	5	57	40
512	K	RG	5	5	28	5
513	K	RG	5	5	80	5
514	K	RG	5	5	28	10
515	K	RG	20	5	40	57
516	K	RG	20	5	28	28
517	K	RG	5	5	113	5
518	K	RG	5	5	10	5
519	K	RG	10	5	80	5
520	K	RG	10	5	40	5
521	L	SR	5	5	5	5
522	L	SR	5	5	57	5
523	L	SR	20	5	80	28
524	L	SR	5	5	40	5
525	L	SR	20	5	453	40
526	L	SR	5	10	20	57
527	L	SR	10	5	57	5
528	L	SR	10	5	160	80
529	L	SR	5	5	14	20
530	L	SR	10	5	80	5
531	M	RG	28	5	226	20
532	M	RG	5	5	40	5
533	M	RG	5	5	20	5

534	M	RG	5	5	20	5
535	M	RG	5	5	10	5
536	M	RG	5	5	20	5
537	M	RG	5	5	5	5
538	M	RG	5	5	5	5
539	M	RG	20	5	57	5
540	M	RG	5	5	40	40
541	M	RG	5	5	5	5
542	M	RG	5	5	5	5
543	M	RG	5	5	5	5
544	M	RG	5	5	20	5
545	M	RG	5	5	5	5
546	M	RG	5	5	20	5
547	M	RG	5	5	5	20
548	M	RG	5	5	40	14
549	M	RG	5	5	14	5
550	M	RG	5	5	20	14
551	N	SR	10	5	40	5
552	N	SR	10	5	40	40
553	N	SR	5	5	20	5
554	N	SR	10	5	5	5
555	N	SR	5	5	5	5
556	N	SR	5	5	14	5
557	N	SR	40	5	320	40
558	N	SR	5	5	5	5
559	N	SR	5	5	5	5
560	N	SR	5	5	14	10
561	N	SR	5	5	40	20
562	N	SR	5	5	5	5
563	N	SR	10	5	40	28
564	N	SR	5	5	28	5
565	N	SR	5	5	5	5
566	O	RG	5	5	5	5
567	O	RG	160	20	320	160
568	O	RG	10	5	40	5
569	O	RG	20	5	57	5
570	O	RG	5	5	5	5
571	O	RG	80	40	453	905
572	O	RG	40	80	160	453
573	O	RG	20	5	113	5
574	O	RG	10	10	80	80
575	O	RG	5	5	40	20
576	L	RG	14	5	57	40
577	L	RG	5	5	80	5
578	L	RG	5	5	20	57
579	L	RG	5	5	5	5
580	L	RG	5	5	20	5
581	L	RG	5	5	28	5
582	L	RG	5	5	5	5

583	L	RG	5	5	5	5
584	L	RG	5	5	14	5
585	L	RG	5	5	20	5
586	L	RG	5	5	5	5
587	L	RG	10	5	20	5
588	L	RG	5	5	5	5
589	L	RG	5	5	5	20
590	L	RG	5	5	20	5
591	L	RG	5	10	5	160
592	L	RG	5	5	28	20
593	L	RG	5	5	20	5
594	L	RG	5	5	5	5
595	L	RG	5	5	5	5
596	L	RG	5	5	20	28
597	L	RG	5	5	40	5
598	L	RG	5	5	5	5
599	L	RG	5	5	5	5
600	L	RG	5	5	20	5
